# Understanding the dog population in the Republic of Ireland: insight from existing data sources?

**DOI:** 10.1186/s13620-022-00223-8

**Published:** 2022-07-14

**Authors:** Simon J. More, Daniel M. Collins, Natascha V. Meunier, Locksley L. McV. Messam, Rob Doyle, Aiden Maguire, Sean Murray, Patricia Reilly, Catherine Lawler

**Affiliations:** 1 UCD Centre for Veterinary Epidemiology and Risk Analysis, School of Veterinary Medicine, University College Dublin, Belfield, Dublin, D04 W6F6 Ireland; 2 School of Veterinary Medicine, University College Dublin, Belfield, Dublin, D04 W6F6 Ireland; 3 Animal Health Ireland, 4-5 The Archways, Carrick on Shannon, Co. Leitrim N41 WN27 Ireland; 4 Department of Agriculture, Food and the Marine, Kildare St, Dublin, D02 WK12 Ireland

**Keywords:** Pet dogs, Dog population, Ireland, Existing databases, National policy

## Abstract

**Background:**

Reliable information about national pet dog populations is an important contributor to informed decision-making, both by governments and national dog welfare organisations. In some countries, there is an improved understanding of aspects of the national pet dog population, but as yet limited published information is available in Ireland. The current study reviews the utility of existing data to inform our understanding of recent changes to the pet dog population in Ireland, including both biological and organisational processes.

**Results:**

Based on national data on dog licencing and microchipping registration, pet dog numbers have remained relatively stable in recent years (ie prior to the COVID-19 pandemic). Since 2015, there has been a substantial decrease in the number of dogs managed through dog control centres. Although the completeness of the data are likely variable, there appears to be substantial, and increasing, number of dogs moving from Ireland to other countries, including UK, Sweden, Italy, Germany and Singapore. We also note an increase (albeit much smaller) in the number of dogs being moved into Ireland.

**Conclusions:**

This study highlights the challenges faced when using existing national data to gain insights into the dog population of Ireland. The linking of existing national databases (individual dog identification, dog licencing, dog control statistics) has the potential to improve both the representativeness and accuracy of information about the Irish pet dog population. In the next phases of our work, we will focus on the work of dog welfare organisations, given both the increased role played by these organisations and the substantial public funding that has been committed in this sector.

**Supplementary Information:**

The online version contains supplementary material available at 10.1186/s13620-022-00223-8.

## Background

Information about national pet dog populations is an important contributor to informed decision-making, both by governments and national dog welfare organisations. For governments, this information can assist both to monitor existing policies (including compliance with existing legislative instruments) [1, 2] and to inform ongoing policy development [3]. It is also important to the work of national dog welfare organisations, with their primary focus on advocacy, action and education [4, 5]. The effectiveness of their work will be maximised if informed by a range of relevant information, such as an ongoing assessment of trends in dog health and welfare indicators [6], a clear understanding of key points of national concern (such as puppy farms and the illegal transportation of dogs, including puppies) [4], and of an ongoing assessment of the impact of advocacy activities.

In a number of countries, considerable efforts are being made to progressively build a robust evidence base in support of an improved understanding of aspects of national pet dog populations.

In Sweden, research has been undertaken over many years with the Agria pet insurance database to investigate mortality and morbidity in the insured dog population. These studies focused on a claims database from a large insurance company. These data are reasonably representative of the national population [7, 8] and covered (during 2016) approximately 38% of the national dog population [9]. Over the years, analysis primarily focused on health-related research questions and included mammary tumours (incidence, prognosis) [10], kidney disease [11] and cruciate ligament rupture [9]. Studies on mortality and survival have also been conducted [12] and the benefits and limitations of using insurance data for companion animal research have been considered in some detail [13].

In Italy, there has been a particular research focus on legislative compliance, noting that it is local authorities that oversee the obligatory requirement for registration and identification of dogs. In a recent study, Carvelli et al. [14] reported that 75.3% of dogs were correctly registered and identified, with these animals more likely to be purebred, neutered, living in urban areas and visiting a veterinarian frequently. This study recommended several strategies to encourage registration, including promoting responsible dog ownership among the general population, engaging with private veterinarians and dog breeders, establishing an effective monitoring system by competent authorities and introducing incentives to enhance dog registrations and fines for owners who do not comply.

In the UK, work has been undertaken by a number of different organisations and research groups, seeking a comprehensive understanding of the national pet dog population. Four broad study approaches have been adopted. Firstly, there are several ongoing studies that utilise existing veterinary practice data. An initiative of the Royal Veterinary College, the Veterinary Companion Animal Surveillance System (VetCompassTM [15]) is investigating a range of health problems in companion animals based on the capture and analysis of data from more than 1800 primary veterinary practices and referral centres in the UK. These analyses form an evidence basis, captured in an extensive portfolio of scientific publications over the last 10 years, across a broad range of health disorders (general, systems-based) [16, 17], prescribing practices [18] and disease surveillance [19]. This approach has since been extended to several countries, including Australia [20, 21]. The University of Liverpool in partnership with the British Small Animal Veterinary Association has established the Small Animal Veterinary Surveillance Network (SAVSNET [22]), which harnesses electronic data on the population of dog owners that visit small animal veterinary practices. SAVSNET seeks to harvest electronic health and environmental data for rapid and actionable research and surveillance, with a current focus on identifying and reporting adverse drug reactions, understanding the needs of dogs as they age, and investigating vaccine hesitancy and strategies to improve vaccine uptake in companion animals [23]. Secondly, Dogs Trust is currently utilising data from multiple existing databases to gain a greater understanding of the spatial density and distribution, demographics and regional trends in size of the UK pet dog population. The study is motivated by key welfare concerns, including the large-scale breeding and sale of puppies from unsuitable environments with regards to health and behavioural development (e.g., puppy farms), and the illegal international transportation of puppies with associated welfare and disease transmission risks (i.e., puppy smuggling) [4]. It relies on the linking, or matching, of data across datasets from a variety of sources, including animal welfare centres, microchipping organisations, pet insurance, local councils, and veterinary practices. Thirdly, several longitudinal cohort studies are underway, seeking a clearer understanding of associations of canine genetics and environment with a range of health and behavioural outcomes [4, 6, 24]. These studies include the Dogslife project, which is following Labrador Retrievers registered with the Kennel Club in the UK [6, 24, 25], and the Generation Pup study, which is focusing on pure- and mixed-breed puppies [4, 26, 27]. Finally, a longitudinal nationwide survey (the Animal Wellbeing (PAW) Project) has been published annually by the People’s Dispensary for Sick Animals’ (PDSA) since 2011, providing an overview of trends and priorities in companion animal welfare in the UK [28]. The 2021 PAW report considers the impact of the COVID-19 pandemic on pets, highlighting concerns relating to pet acquisition, behavioural problems and unscrupulous breeding [29]. Over several reports, PDSA also highlighted obesity in pet dogs and breeding for exaggerated conformation (including brachycephaly) as ongoing welfare concerns. The report highlights the value of pre-purchase consultations to prospective dog owners to address several key issues identified by veterinarians, including ‘welfare at breeding establishments (e.g. puppy farms)’ and ‘poor choice of breed for owner lifestyle’ [29].

To date, there has been little information available in the peer-reviewed literature on the pet dog population in the Republic of Ireland (subsequently referred to as Ireland, or Irish when used as an adjective). With respect to population demographics, an estimated 35.6% of Irish households owned one or more pet dogs in 2007, with 47.3% of them neutered [30, 31]. At that time, more pet dog owning households were found per square kilometre in cities and in the east of the country than in rural areas and in the west. Dog ownership was associated with location, house type, household social class, household composition, the presence of children in the household, and the presence of a cat. In a recent study relating to community-dwelling adults aged 50 years and over, dog ownership was highest among adults aged 50–64 (51%) and lowest among adults aged 75 and over (25%) [32]. Further, the proportion of rural dwellers (49%) owning a dog was almost twice that of Dublin dwellers (26%).

Several recent studies have considered aspects of Irish legislation and controls relating to pet dogs. In a review of records generated by the Cork County Council dog control service during 2007 [2], almost three quarters of official dog control duties related to dogs that were unwanted and/or were not under the control of the owner. The most frequent reasons for a service request included collecting a stray dog from a person’s property, an owned dog being out of control in a public place, and bite incidents or reports of aggressive behaviour. In a review of hospital records-based dog bite injuries in Ireland, Ó Súilleabháin [33] raised concern about use of breed-specific legislation, as currently applied in Statutory Instrument (SI) No. 442/1998 (the Control of Dogs Regulation 1998) [34]. Recently, Keogh et al. found low levels of awareness among the general public (both dog owners and non-dog owners) that key responsibilities of dog owners are prescribed under Irish law. These included the responsibilities of dog identification, prevention of dog straying, abandonment and tail docking and the safeguarding of a dog’s health [1].

In recent years, particularly in the popular press, there have been numerous reports of change in the Irish dog population. There has been an increased role for dog welfare organisations [35], including for the direct surrendering of dogs. A drop in the number of stray dogs euthanised each year has been reported [36]. Further change has been reported in association with the COVID-19 pandemic, including an initial surge in dog ownership [37], followed more recently by the surrendering of unwanted dogs to animal welfare organisations [38] and dog control centres [39], in part as a consequence of behaviour-related problems [37]. As yet, these changes have not been reflected in the Irish scientific literature.

A multi-study research programme is currently underway in Ireland to broaden the evidence base on the national pet dog population. This research will inform a review of the current ex gratia funding model for animal welfare organisations and the underlying public policy objectives is outlined in the national Department of Agriculture, Food and the Marine (DAFM) Animal Welfare Strategy, 2021–2025 [40].

The current study reviews the usefulness of existing data sources to inform our understanding of recent changes to the pet dog population in Ireland, including those relating to biological (demographics, flows, trends) and organisational (the roles of different organisations, regulatory and non-regulatory impacts, drivers of supply and demand) processes. Further, we present a proposal to improve both the representativeness and accuracy of information about the Irish pet dog population.

This is the first output of this multi-study research programme. In the next phases of our work, we will focus on the work of dog welfare organisations, given both the increased role played by these organisations and the substantial public funding that has been committed in this sector.

## Methods

### Definitions

*Dog control centres* operate under The Control of Dogs Act 1986 [41]. Under this Act, each local authority is required to ‘establish and maintain one or more shelters for dogs seized, accepted or detained under any of the provisions of this Act and may, with the consent of the Minister, enter into arrangements with any person for the provision and maintenance of such shelters and for the exercise by such person of the functions of the local authority under this Act in respect of the acceptance, detention, disposal and destruction of stray and unwanted dogs.’ The national Department of Rural and Community Development (DRCD) is the relevant government department, which has responsibility for policy and legislation regarding dog control and dog breeding establishments.

*Dog welfare organisations* are voluntary organisations. National animal welfare policy is overseen by DAFM [40], and funding from DAFM Animal Welfare Grants is available to registered animal welfare organisations to assist in delivery of animal care and animal welfare services [42].

### Background activities

Several activities were conducted to inform later aspects of the work:

- Legislation relevant to dog controls in Ireland were described.
- A conceptual diagram of the Irish dog population was created to represent the various sub-populations of dogs (pets, commercial etc.), locations and organisations which house them (dog control centres, dog welfare organisations) and the movement of dogs between these groupings as well as into and out of Ireland. The diagram was informed by the expert opinion of co-authors.
- The location of dog welfare organisations that were supported financially by DAFM in 2020 and of the dog control centres was mapped using ArcView (version 3.2). In Ireland, the location of each business and private premises may be derived from the Eircode (the Irish postcode system [43]), which is unique to each premises. Past funding support from DAFM to animal welfare organisations was determined based on publicly available data, for December 2016 [44], December 2017 [45], December 2018 [46], December 2019 [47] and December 2020 [48].

### The Irish pet dog population

The following sources of data were used to gain an understanding of dog population numbers in Ireland:

- *Dog licencing.* These data are collated by AnPost which gives access to each local authority within the DRCD. These data, covering the periods 2000–2020, are publicly available [49], as is licence pricing information [50].
- *Dog microchip and identification.* Microchip and identification data are collected by four commercial companies: Animark [51], Fido [52],the Irish Kennel Club [53], and Microdog ID Ltd. [54]. Each company is registered in compliance with conditions set down in the Microchipping of Dogs Regulations 2015 (S.I. No. 63/2015) [55].
- *Population estimates* for Ireland from the European pet food industry (Fediaf) Facts & Figures [56]. However, the method used to estimate the Irish dog population is not available.
- *Dog control centres.* Annual statistics relevant to dog control centres in Ireland are collated by each individual local authority within the DRCD [49].

Approaches were also made to Veterinary Ireland (the representative body for veterinary surgeons in Ireland) and several pet insurance companies, but no data relating to dog numbers were made available.

Although private dog sales data are not regularly collected, a proportion of these sales will be advertised online on public listings. Therefore, a pilot study was undertaken during a 6-month period (01 September 2021 to 28 February 2022) of dog sales advertised on Irish websites to determine the potential utility of these data to provide insights into population numbers and dynamics. We restricted the pilot study to two websites for dog sales [57, 58], these being those ranked highest by Google for “dog sales Ireland” on 02 December 2021. We judged that it was both legal and ethical to web-scrape these sites, noting that these data are online and publicly available, the data were to be used for research purposes only, and web-scraping was not prohibited on either site in the stated terms and conditions or based on the robots exclusion standard. No personal information on the sellers was collected. During this 6-month period, advertisements were downloaded on an ongoing basis. For each advertisement, information was collected on the data published, price, breed, date of birth, current age, number of dogs for sale, number of females/males, microchip number, colour, temperament, location, whether the dog was registered with the Irish Kennel Club (IKC), whether the dog was de-wormed, vaccinated or neutered, whether the seller was registered with DAFM, and the maximum number of breeding bitches owned. Advertisements were cleaned using R software (rvest and httr packages). There was expected duplication in the dataset, with the potential for sellers to relist the same advertisements, to create new advertisements for the same dogs, or to create an advertisement for another website. There was an increase month by month as more advertisements were captured and the likelihood of recording the duplicates increased. Duplicate advertisements were identified based on identical microchip numbers and birth dates, and only the first instance of any advertisement was retained. Descriptive analyses were conducted to determine the number of listings (each month, by sex and age), the percentage of dogs de-wormed, vaccinated, neutered and IKC registered, the range in prices, the breed, and the distribution of sellers by maximum number of breeding bitches.

### The movement of dogs to and from Ireland

A range of data were used to gain an understanding of the dynamics of the dog population in Ireland:

- *Pet passports.* The EU system of passports for pets allows cats, dogs and ferrets to travel between EU Member States and some other countries that are part of the scheme [59]. Therefore, when leaving or entering Ireland, all dogs must have an individual pet passport regardless of whether the reason for travel is commercial or not (eg. for pet movement). Pet passports are issued by DAFM to private veterinary practitioners PVPs and dog welfare organisations who subsequently issue them to pet dog owners [60]. The pet passport database, maintained by DAFM, records the annual number of pet passports issued to PVPs and dog welfare organisations, but provides no information on the number that were subsequently issued to pet dog owners. The DAFM database was interrogated as part of this study.
- Some official and commercial data are available on the incoming and outgoing movement of dogs.
  - ◦ *Data from the European Commission.* Trade Control and Expert System (TRACES) is the online platform of the European Commission to facilitate sanitary and phytosanitary certification of animals, animal products, food, feed and plants, for importation into the EU, for intra-EU trade and for export out of the EU [61]. These data are limited to movements where certification is required (i.e., commercial non-pet movements). The TRACES database was interrogated as part of this study.
  - ◦ *Data from commercial enterprises.* Airline and ferry companies were contacted, seeking data on incoming dog movements, and the reported data is as notified to DAFM. Outgoing dog movement data were not available.

### Data analysis

Using R software version 4.0.3, simple graphs were constructed for all measures to aid in the assessment of trends.

### A qualitative review of data quality

A qualitative review of each of the data sources used in this study was undertaken, including a description of the context in which these data are collected and an assessment of data quality, including characteristics of the information, representativeness of the national dog population, accuracy of the information (whether the values stored for an object are the correct value) and suggestions to address concerns raised.

## Results

### Relevant legislation

There is a range of legislation relevant to the control of dogs in Ireland:

- *The Control of Dogs Act 1986* [41] requires dog owners to purchase a licence for all dogs older than 4 months of age (or once the puppy is removed from its dam or foster mother, if younger). The licence applicant must be older than 16 years old, and pet owners may purchase either an annual dog licence (€20) or a lifetime dog licence (€140). Kennel owners with multiple dogs (unspecified numbers) are required to purchase a general dog licence direct from their local authority (€400). During the online purchase of a dog licence, although there is an opportunity to enter microchip details, registration can proceed without this information.
- *Statutory Instrument (S.I.) No. 442/1998 Control of Dogs Regulation 1998* [34] places restrictions on certain breeds of dogs, relating to leashing, muzzling and identification of ownership.
- *The Dog Breeding Establishments Act 2010* [62] regulates dog breeding establishments and anyone keeping 6 or more intact bitches.
- *S.I. No. 602/2014 Pet Passport (No. 2) Regulations 2014* [60] implements Regulation (EU) No. 576/2013 [63] on the rules applicable to the non-commercial movement of a pet dog, cat or ferret, which accompanies its owner during his or her movement. Where the pet animal is being moved for the purposes of sale or change of ownership, the animal must meet the EU animal health requirements applicable to trade in or imports into the Union of animals of the species concerned (‘commercial movement’).
- *S.I. No. 63/2015 Microchipping of Dogs Regulations 2015* [55] requires all dogs to be microchipped by 12 weeks of age, or sooner if moved from place of birth. The microchip must comply with ISO Standard 11,784 and be legible with devices compatible with ISO Standard 11,785. The dog identification database must include details of the unique identification, owner, identity of person performing the microchipping and the death of dog. In addition, the database operator must be a full member of EuroPetNet [64].
- *S.I. No. 681/2019 Animal Health and Welfare (Sale or Supply of Pet Animals) Regulations 2019* [65] regulates the sale or supply of pet animals, including the requirement that all advertisements of sale must include a dog’s microchip number. Under this S.I., a pet animal is defined as an animal kept, or intended to be kept, by a person as a pastime or hobby, for companionship and/or for ornamental purposes, and does not include a farm animal. The legislation is applicable to sellers/suppliers of 6 or more pet animals in a calendar year, including animal homing organisations and commercial entities, but not to farm animals, establishments covered under the Dog Breeding Establishments Act 2010 [62], or facilities run by, or on behalf of, local authorities.

### Conceptual diagram

**Fig. 1:**
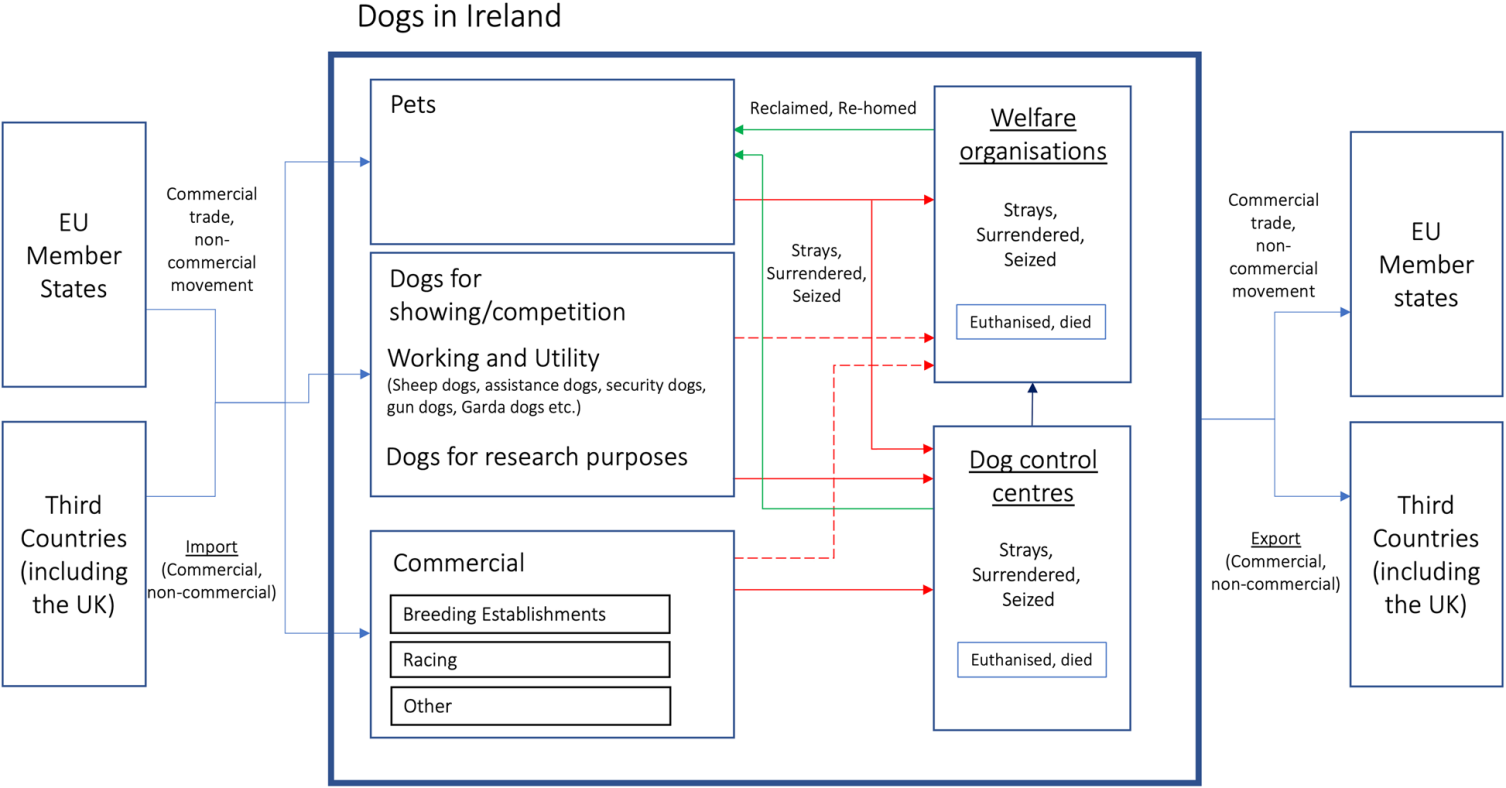
A conceptual diagram of the dog population in Ireland, including different subpopulations (pets, commercial, dogs for other purposes), organisations (dog control centres, dog welfare organisations), and the flows of dogs both between these groupings and to/from Ireland

A conceptual diagram of the dog population in Ireland, and of the movement of dogs into and out of Ireland, is presented in Fig. 1. The diagram highlights the movement of dogs from and to other EU Member States and Third Countries (including the UK). Within Ireland, there is a flow of dogs between different subpopulations (pets, commercial, dogs for other purposes) and different organisations, including dog control centres and dog welfare organisations.

### Animal welfare organisations and dog control centres

**Fig. 2:**
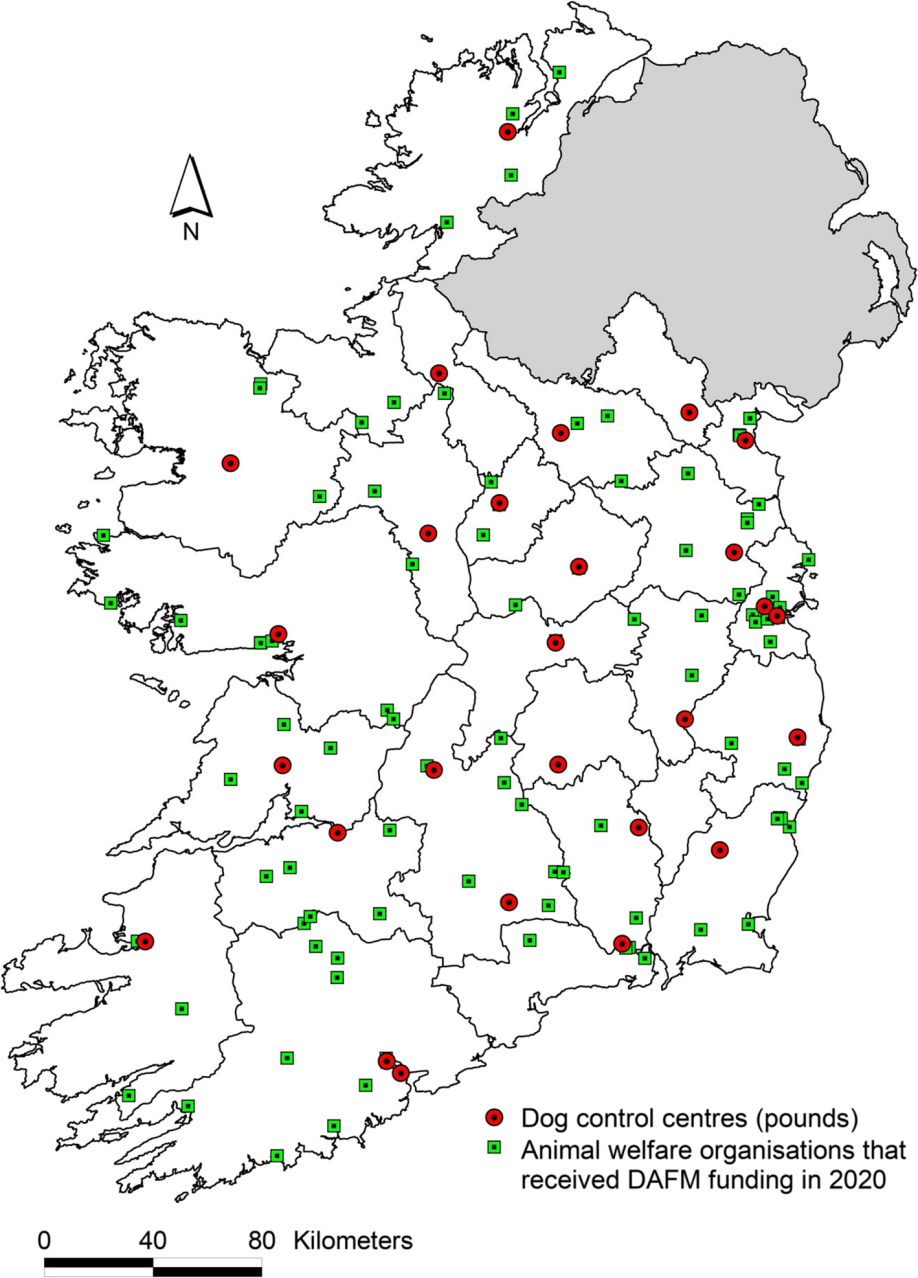
The location of 27 dog control centres and 72 funded dog welfare organisations in Ireland. The 72 dog welfare organisations each received funding in 2020 under the DAFM Animal Welfare Grants to registered animal welfare organisations to assist in delivery of animal care and animal welfare services

Dog control centres and dog welfare organisations are located throughout Ireland (Fig. 2). There are currently 27 dog control centres. During 2016 to 2020, DAFM provided €13.9 million to animal welfare organisations, as presented in Table 1. In 2020, 101 animal welfare organisations received funding, including 72 dog welfare organisations.

**Table 1:** Grants provided to animal welfare organisations by the Department of Agriculture, Food and the Marine from 2016 to 2020

Funding date	Number of animal welfare organisations that received animal welfare grants				Total value of grants provided to animal welfare organisations
	Handling dogs only	Handling dogs and other species	Handling other species only	Total	
December 2016	11	65	61	137	€2,460,500
December 2017	14	57	40	111	€2,560,000
December 2018	12	62	34	108	€2,751,000
December 2019	8	57	41	106	€2,906,000
December 2020	7	65	29	101	€3,200,000
Total					€13,877,500

### The Irish pet dog population

#### Dog licences

**Fig. 3:**
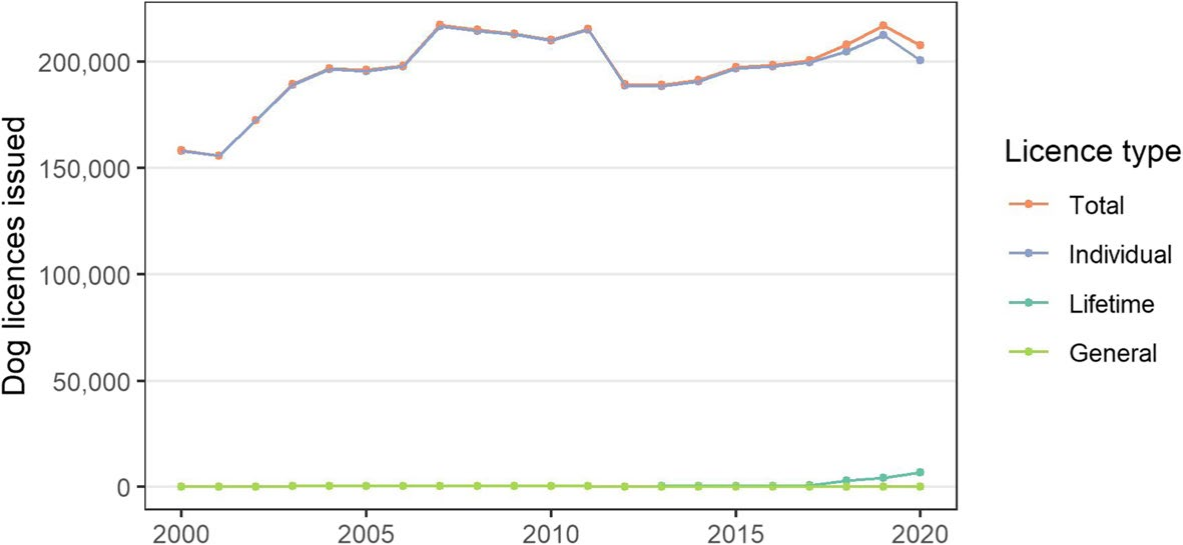
The number of dog licences issued in Ireland during 2000–2020, by type of licence. These data were collated by the Department of Rural and Community Development and are available at https://www.gov.ie/en/collection/879d4c-dog-control-statistics/

The number of dog licences issued in Ireland during 2000–2020 is presented in Fig. 3 (also in Table S1 in the Supplementary material). Note the drop off in licences issues from 2011 to 2012, and in 2020. There was an upward trend in dog licences issued, increasing by 31.2% (49,424) over the 20-year period since 2000. The majority of this increase was prior to 2007.

#### Microchipping

**Fig. 4:**
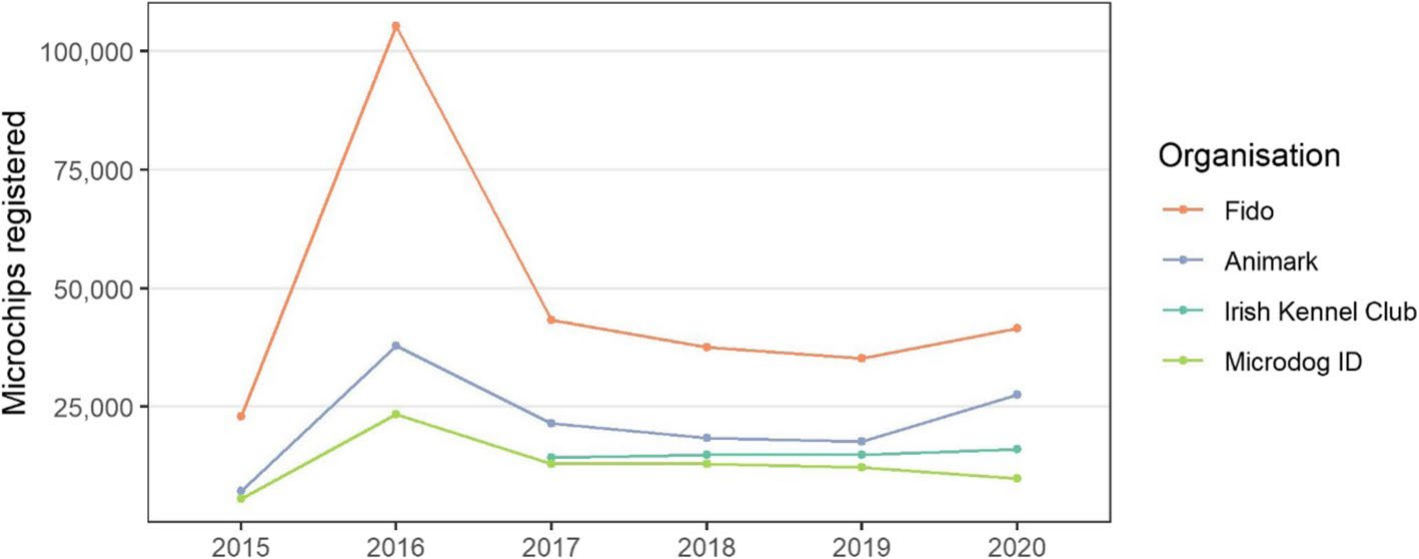
The number of dog microchips registered in Ireland by Animark, Fido and Microdog ID from 2015 to 2020 and by the Irish Kennel Club from 2015 to 2020

The number of dog microchips issued in Ireland by Animark, Fido and Microdog ID from 2015 to 2020 and by the IKC from 2017 to 2020 is presented in Fig. 4 (also in Table S2 in the Supplementary material). There was a very substantial uptake of microchipping in 2016 (noting the legal requirement for mandatory microchipping in Ireland from 01 June 2015 [55]), although the number has been relatively stable since. The mean number of microchips since 2017 is 87,787 per year.

#### European pet food industry (Fediaf) population estimates

**Fig. 5:**
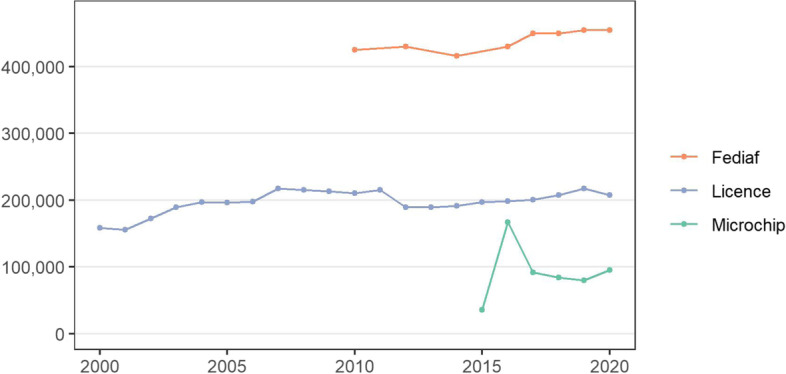
The total number of dog licences issued and microchips registered per year in Ireland, during 2000–2020 (where available). The population estimates for the domestic dog population in Ireland from the European pet food industry (Fediaf) are also presented

During 2010–20 (except 2011, 2013 and 2015), Fediaf estimates were available of the number of dogs in Ireland, and the percentage of households owning at least one dog. These estimates included 425,000 dogs and 26% households owning at least one dog in 2010, 430,000 and 20% in 2012, 416,000 and 20% in 2014, 430,000 and 22% in 2016, 450,000 and 34% in 2017, 450,000 and 34% in 2018, 455,000 and 35% in 2019, and 455,000 and 25% in 2020. The Fediaf population estimates follow an upward trend, with an increase of 39,000 dogs (9.4%) from 2014 to 2020. The same period saw a similar increase in dog licence registrations of 16,496 (8.6%) (Fig. 5).

There is no visual relationship between the number of dog licences and microchip registrations per year (Fig. 5). The dog licences are, for the most part, individual licences that need to renewed annually whereas the microchip registrations are once-off.

#### Dog control centres

**Fig. 6:**
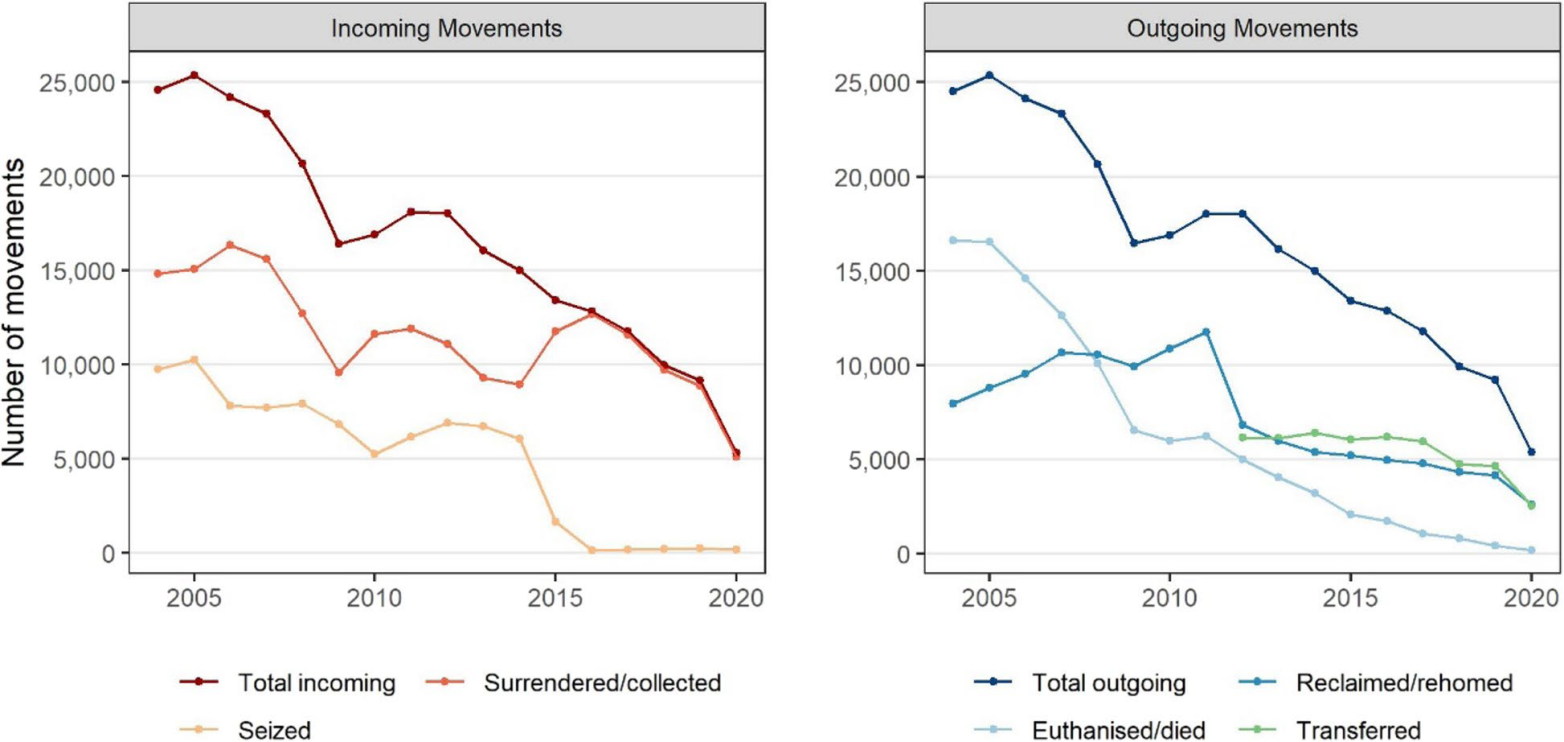
Incoming and outgoing movements from dog control centres in Ireland from 2004 to 2020. These data were collated by the Department of Rural and Community Development and are available at https://www.gov.ie/en/collection/879d4c-dog-control-statistics/

Annual statistics relevant to dog control centres in Ireland during 2004–2020 are presented in Fig. 6 (also in Table S3 in the Supplementary material). There have been decreasing trends in both the incoming and outgoing movement of dogs from dog control centres over the last 15 years. In any given year, the number of dogs moving into these centres (surrendered, collected or seized) roughly equals those leaving (reclaimed, rehomed, euthanised, died or transferred). From a peak of incoming: outgoing movements of 25,332:25,364 in 2005, these have decreased to the most recent figures of 5310:5371 in 2020 (that is, a 78.4% decrease in incoming movements and a 78.1% decrease in outgoing movements during 2005 to 2020). There is a small peak in intake from 2011 to 2012 which aligns with the decrease in licence registrations that was observed in Fig. 3.

#### Online private dog sales

During a six-month period (01 September 2021 to 28 February 2022), there were 5201 unique advertisements representing 14,732 dogs (a mean of 2.8 dogs per advertisement, median 2, maximum 14). A mean of 28.7 (median 27) advertisements were added each day, with a maximum of 78 added on 24 September 2021.

Of the 14,732 dogs advertised, the median age was 69 days and there were 8815 (55%) males. More than 92% (*n* = 13,602) of dogs advertised were less than 4 months old at the time of the listing. Based on the information presented, 99.2% (*n* = 14,620) of dogs were de-wormed, 99.1% (*n* = 14,598) were vaccinated, 1.2% (*n* = 183) were neutered, and 27.6% (*n* = 4061) were IKC registered. The advertised mean sale price of dogs was €830 (median €750, maximum €6500), and the most frequent breeds were Poodle crosses (449 advertisements, including Doodles (164), Cockapoos (183) and Cavapoos (102)), Labrador Retrievers (375), German Shepherds (293), Golden Retrievers (258), Jack Russell Terriers (208), Cocker Spaniels (203) and Collies (202).

There were 69 identified breeders that provided a registration number for a dog breeding establishment. These 69 breeders were associated with 381 advertisements, being 7.3% (381/5201) of all advertisements. The top 3 sellers had 44, 21 and 18 listings, and were seeking to sell 210, 47 and 52 dogs, respectively. Among the sellers who specified the number of maximum breeding bitches on their premises, there were 3 sellers with a maximum of 1–10 breeding bitches, 34 with 11–12, 12 with 21–30, 3 with 31–40, 1 with 41–50, 3 with 51–50, 11 with 61–100, 1 with 101–180 and 1 with 181–300.

### The movement of dogs to and from Ireland

#### Pet passports issued

The number of pet passports issued by DAFM to PVPs and dog welfare organisations in Ireland during 2014–20 is presented in Table 2. There was a 15.7% decrease in passports issued by DAFM between 2017 and 2020.

**Table 2:** The number of pet passports issued by the Department of Agriculture, Food and the Marine to private veterinary practitioners (PVPs) and animal welfare organisations in Ireland during 2014–20. These data may not reflect the actual number of pet passports issued by PVPs and animal welfare organisations to pet dog owners

	2014	2015	2016	2017	2018	2019	2020	Total
*Private veterinary practitioners*	11,836	22,208	18,391	19,450	18,418	18,400	18,281	126,984
*Animal welfare organisations*	2855	4512	4612	5462	3799	3570	2709	27,519
Total	14,691	26,720	23,003	24,912	22,217	21,970	20,990	154,503

#### Dog movements based on data from the European Commission

A summary of the official record of dog movements from Ireland to other European Economic Area (EEA) countries and to third (ie non-EU) countries during 2016–20 is presented in Table 3 and Table S4 (in the Supplementary material), respectively. During this period, records are available for 55,240 exported dogs, with the most frequent destinations including the UK (41,167 dogs), Sweden (6457), Italy (1874), Germany (1583) and Singapore (1290).

**Table 3:** The number of dog movements from Ireland to other European Economic Area countries during 2016–20, as recorded in TRACES, including those dogs en-route to third countries (Argentina, Bermuda, Singapore, United States). TRACES is the online platform of the European Commission to facilitate sanitary and phytosanitary certification of animals, animal products, food and feed and plants, into the EU, for intra-EU trade and EU exports (https://ec.europa.eu/food/animals/traces_en)

Country	2016	2017	2018	2019	2020	Total
European Union countries						
Austria	5	2	1	2	5	15
Belgium	47	63	33	39	50	232
Bulgaria	–	15	–	–	–	15
Croatia	–	–	1	–	–	1
Cyprus	–	1	–	–	–	1
Czechia	63	45	114	90	108	420
Denmark	12	5	3	3	6	29
Finland	25	26	24	25	5	105
France	15	27	38	26	23	129
Germany	336	340	364	256	287	1583
Greece	19	–	–	–	–	19
Hungary	–	–	–	1	2	3
Ireland	18	–	–	–	2	20
Italy	443	384	336	341	370	1874
Latvia	–	–	–	–	1	1
Lithuania	–	1	1	–	–	2
Luxembourg	3	–	–	–	–	3
Netherlands	20	35	102	79	32	268
Poland	1	4	4	1	5	15
Portugal	69	38	7	48	24	186
Slovakia	–	–	–	–	1	1
Slovenia	11	4	3	8	19	45
Spain	66	79	46	56	51	298
Sweden	1201	1183	1400	1418	1255	6457
Non-European Union countries						
Argentina (via Spain)	1	–	–	–	–	1
Bermuda (via England)	1	–	–	–	–	1
Gibraltar	1	–	–	–	–	1
Norway	50	69	52	19	5	195
Singapore (via Germany)	–	–	–	4	7	11
Switzerland	12	17	8	8	5	50
United Kingdom^a^	9625	10,571	7810	7368	5793	41,167
United States (via Germany or England)	–	–	–	19	28	47
Total	12,044	12,909	10,347	9811	8084	53,195

^a^ The United Kingdom left the European Union on 31 January 2020

A summary of the official record of dog movements from other EU countries to Ireland from January 2018 through to July 2021 is presented in Table 4. There was a large increase in dogs moving into Ireland in 2021, compared to previous years during this period, notably from Hungary (438 during the first 7 months of 2021 compared with an annual mean of 170 during 2018–2020), Poland (255, 51) and Romania (116, 59.3).

**Table 4:** The number of dog movements from other EU countries into Ireland during 2018 through to July 2021, as recorded in TRACES, which is the online platform of the European Commission to facilitate sanitary and phytosanitary certification of animals, animal products, food and feed and plants, into the EU, for intra-EU trade and EU exports (https://ec.europa.eu/food/animals/traces_en)

Country	2018	2019	2020	2021 (Jan-Jul)	Total
European Union countries					
Belgium	1	–	4	6	11
Croatia	3	1	12	35	51
Cyprus	1	2	2	1	6
Czech Republic	7	1	9	15	32
Estonia	–	–	–	1	1
Finland	1	–	–	1	2
France	–	2	2	10	14
Germany	–	4	4	28	36
Greece	–	1	–	1	2
Hungary	157	157	196	438	948
Ireland	–	–	2	–	2
Italy	–	1	6	12	19
Latvia	–	1	1	8	10
Lithuania	1	6	12	62	81
Malta	1	–	–	–	1
Poland	29	47	77	255	408
Portugal	–	–	–	7	7
Romania	98	26	54	116	294
Slovakia	–	–	3	30	33
Slovenia	–	–	–	2	2
Spain	1	88	1	54	144
Non-European Union countries					
United Kingdom^a^	2	17	4	–	23
Total	302	354	389	1082	2127

^a^ The United Kingdom left the European Union on 31 January 2020

#### Dog movements based on data from commercial enterprises

As reported to DAFM, the number of dogs recorded on commercial flights into Dublin, Shannon and Cork from January 2015 to June 2020 is presented in the Supplementary material (Tables S5 and S6). The number of dogs recorded on commercial ferries into Cork Roscoff during July to October 2020, into Cork Ringaskiddy from January to February 2020, and into Rosslare, Co. Wexford from 2018 to May 2021 is presented in the Supplementary material (Tables S7 to S9, respectively). In 2020, 1124 dogs were recorded on commercial flights and 1947 dogs on commercial ferries into Rosslare.

### The quality of available data

**Fig. 7:**
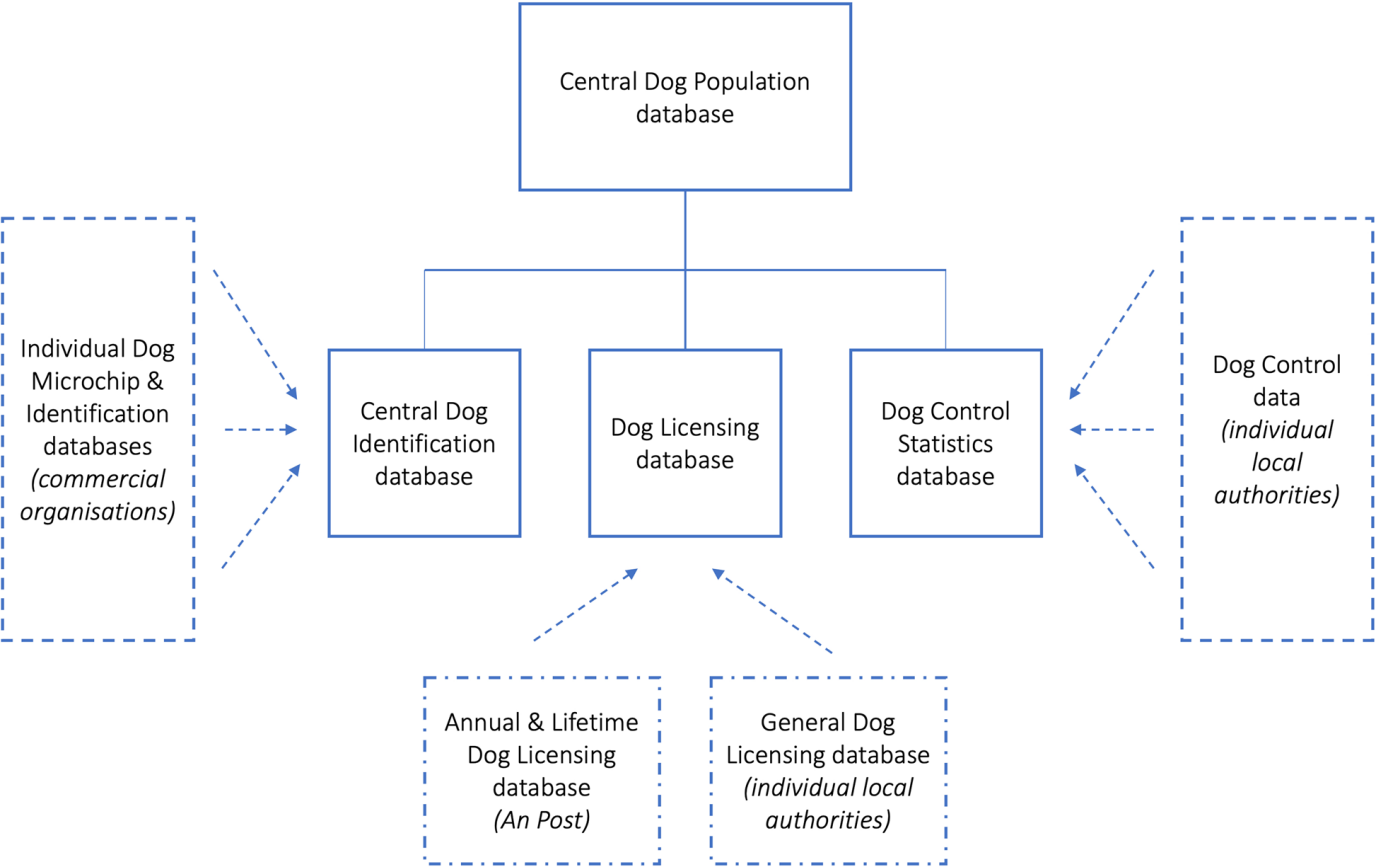
A proposal to improve both the representativeness and accuracy of information about the Irish pet dog population by linking existing national databases

A brief description and evaluation of existing potential data sources for estimating the Irish pet dog population and the movement of dogs to and from Ireland is presented in Table 5. Relevant to the Irish dog population, the representativeness of existing data sources were considered either low (dog licencing data, dog control statistics) or unknown (dog microchipping and identification data), and the accuracy of information considered either uncertain (dog licencing data), variable (dog microchipping and identification data) or likely variable (dog control statistics). Relevant to the movement of dogs to and from Ireland, the representativeness of all existing data sources (pet passport data, dog movements data (from the European Commission, from commercial enterprises)) were considered low, whereas the accuracy of information was considered very high (dog movements data (from the European Commission), low (pet passport data) or very low (dog movements data (from commercial enterprises)). Linked with the suggestions in Table 5, we present a proposal to improve both the representativeness and accuracy of information about the Irish pet dog population by linking existing key national databases (Fig. 7).

**Table 5:** Brief description and evaluation of existing potential data sources for estimating the Irish pet dog population and the movement of dogs to and from Ireland

Data source	Description	Assessment of quality
A. The Irish pet dog population		
Dog licencing and registration database	Legislative requirement for dog owners to purchase a dog licence either for individual dogs greater than 4 months of age (annual, €20; lifetime, €140) or for multiple dogs (general, €400 each year). Annual and general licences must be renewed yearly. Data is collected by An Post which gives access to each local authority within the Department of Rural and Community Development. *Data collected:* • Applicants: Name, age, address, Eircode, phone number and e-mail. • Dog: Name, sex, microchip status and number if present, colour and breed.	*Characteristics of information:* Database contains information on the annual number of: • New individual dog licences issued. • Individual dog licences renewed. • Lifetime dog licences issued (for the duration of the dog’s life). • General licences (for multiple dogs) issued. • General licences (for multiple dogs) renewed. *Representativeness of dog population*: Low. a. Not a one-to-one relationship between number of licences issued and number of dogs in Ireland since i. No way to know if licence non-renewal represents non-compliance or death of dog as death information is not collected. ii. The number of dogs for which a general licence applies is not recorded. iii. A priori excludes dogs younger than 4 months old, dogs housed by the Irish Society for the Prevention of Cruelty to Animals (ISPCA) or Gardaí, currently possessed by a local authority, sight dogs for the blind and dogs imported into the State for less than 30 days. b. Compliance is not enforced and percentage compliance unknown. *Accuracy of information*: Uncertain. • Data provided by applicant’s self-report, no verification of accuracy of dog(s) date of birth, sex, breed or address (mailed in or online). • No microchip requirement for dog(s) prior to licence application. Identity of dog cannot be determined. *Suggestion:* • Requirement of microchip number prior to issuing of licence. • Linkage with the following data sources. • Dog identification data (via microchip number). • Local authority dog control statistics.
Dog microchipping and identification data	Legislative requirement that all dogs be micro-chipped and registered in a dog identification database by 12 weeks of age, or before they leave the property on which they are born (whichever comes earlier). Currently in Ireland, there are four DAFM-approved microchip databases, each sharing data with EuroPetNet (http://www.europetnet.com). *Data collected:* • Applicant (or possessor of dog): Name, address, contact details. • Dog: Date of birth, breed, colour and markings, sex, address of premises of residence, microchip number and date of insertion, date and cause of death (if relevant), date lost (if relevant). • Additional information: Identity of person inserting microchip.	*Characteristics of information:* Four databases give yearly and total information on the dogs that have had a microchip inserted and have been registered. *Representativeness of dog population*: Unknown. • Compliance not enforced. Percent compliance unknown. *Accuracy of information:* Variable. • Microchip information - likely high as this can only be undertaken by specified trained individuals • Identification of person claiming ownership/possession – potentially high - Requires official identification (passport, driver’s licence or identification issued by the Gardaí [the Irish police]) but depends on enforcement. • Date of birth, colour, breed, sex of dog and address of dog – likely high - Microchip insertion done in the context of veterinary clinics done by a veterinary surgeon, veterinary nurse or another trained professional. • Information on death and/or loss of dog - Uncertain - this depends on owner compliance. • Change in ownership – not enforced or recorded automatically. • Duplication – dog might be registered in more than one of the databases under different names due to change of ownership, lack of updating and non-linkage between them. *Suggestion:* • The four dog identification databases should be linked to each other or to a central government database resulting in each microchip number being linked to one and only one Irish owner/address. • The dog licensing database should be linked to the dog identification databases to ensure consistency and verification of recorded information. • The dog identification should be linked with local authority databases which contain information on dogs strayed, surrendered and seized, rehomed, euthanised etc.
Dog controls statistics	These data are published annually by the Department of Rural and Community Development (https://www.gov.ie/en/collection/879d4c-dog-control-statistics/). *Data published:* • Numbers of strayed, surrendered and seized dogs. • Numbers of dogs reclaimed, rehomed, and transferred to welfare groups. • Number of dog deaths (including euthanized dogs).	*Characteristics of information* Database contains information on the annual number of dogs that are possessed by the local authorities including information of the movement of dogs in and out of the local authorities. Thus, it provides information on pet dogs not under the control of a private owner: • The number of stray, surrendered and seized dogs. • The number of re-homed/reclaimed dogs. • The number of dogs transferred to welfare organisations. *Representative of dog population:* Low, but likely possibly representative of pet dog population not under control of private owners. *Accuracy of information:* Likely variable. • Dependant on accuracy of data gathering mechanisms of local authorities. • Dependant on timeliness of data gathering mechanisms of local authorities.
	*Data published:* • Individual dog licence. • A lifetime licence (for the duration of the dog’s life). • A general licence (for multiple dogs).	*Characteristics of information:* Database contains information on the annual number of: • Individual dog licences (online and in person) issued. • Lifetime dog licences issued (for the duration of the dog’s life). • General licences (for multiple dogs) issued. *Representativeness of dog population and accuracy of information:* See previous comments on dog licensing database. *Suggestion:* • Link database to both dog identification and dog licensing databases. This will enhance the accuracy of the dog identification and dog licensing databases as this will allow one to determine and update both with regards to deaths and losses of dogs.
B. The movement of dogs to and from Ireland		
Pet passport data	In accordance with Regulation (EU) No 576/2016: 1. Each dog must be identified by a microchip, or by a tattoo applied before 3 July 2011. 2. A valid EU pet passport, issued by a private veterinary practitioner (PVP), is required for movement of dogs throughout the EU. Passports are required both for commercial and non-commercial movements of dogs into and out of Ireland.	*Characteristics of information:* This database records the annual number of pet passports issued to private PVPs yearly. *Representativeness of dog population:* Low. • The number of passports issued to PVDs is not a reflection of the number of passports issued by the PVP. *Accuracy of information:* Low. • No information in the database on the dogs for which passports have been issued. *Suggestion:* • Request data from private veterinary practitioners on dogs for which pet passports have been issued monthly. At a minimum, the microchip number of each dog issued a passport should be reported to DAFM.
Dog movements data (from the European Commission)	TRACES (Trade Control and Expert System) is the online platform of the European Commission to facilitate sanitary and phytosanitary certification of animals, animal products, food and feed and plants, into the EU, for intra-EU trade and EU exports.	*Characteristics of information* These data capture the yearly number of dogs that are commercially exported to EU member States. *Representativeness of dog population:* Low, as data reflects movements where certification is required, including the commercial movement of dogs between EU Member States (e.g., private operators or charities selling or supplying dogs for re-homing). Thus, it is representative for that subset of dogs requiring certification. • Does not capture pets travelling with owners from Ireland to another EU Member State because animal health certification is not required. • Inaccurate to the extent that certification might be required but not sought. *Accuracy of information:* Very high, as dogs registered on the TRACES database must have a microchip and a pet passport and must come from establishments registered with DAFM.
Dog movements data (from commercial enterprises)	Data are collected by commercial organisations, including airlines and ferry companies, as part of their commercial operations. These data are not linked to TRACES.	*Characteristics of information:* The data contain heterogeneously collected records of inward movements of dog to Ireland. *Representativeness of dog population:* Low. • Reporting reliant on owner compliance. • Reporting varies with the commercial operator. • Reporting various with port of entry. Further, commercial and non-commercial movements are not distinguished. These data relate solely to inward movements. *Accuracy of information:* Very low. • Entirely reliant on owner compliance. • Not verified. *Suggestion:* • Requirement that all dogs entering the country be declared at the port of entry.

## Discussion

The current study was conducted to investigate the utility of existing data to inform our understanding of recent changes to the pet dog population in Ireland, including those relating to biological (demographics, flows, trends) and organisational (the roles of different organisations, regulatory and non-regulatory impacts, drivers of supply and demand) processes. By extension, we hoped to gain insights into aspects of the national pet dog population, and to highlight strengths and areas of concern with respect to the use of existing data for this purpose.

Available data provide fragmented and inaccurate insight into the pet dog population of Ireland. These data are unsuited for estimating the overall size of the total pet dog population, with the only direct information coming from published Fediaf estimates, for which the underpinning data gathering method(s) are unknown. Methods are available to estimate overall dog population size, but their application would require carefully  designed and planned study [66]. The national data do provide hints of several temporal trends, both in terms of biological and organisational processes.

Over the last 20 years, but particularly prior to 2007, there was an upward trend in dog licences issued (Fig. 3), now representing approximately 200,000 dogs licenced annually (Fig. 3). Concurrently, in recent years there has been a relatively stable number of microchips registered annually (approximately 90,000 microchips, Fig. 4). In the popular press, there has been considerable commentary about recent changes to the national pet dog population (including [36, 37, 39]), particularly in relation to the COVID-19 pandemic. Unfortunately, the current study does not clarify this discussion, as apart from limitations of the data available, our study period only partially overlaps with these recent events. We note several points of caution when interpreting these temporal trends in the data. In Ireland, the dog licensing and microchipping databases are currently not linked, which precludes the ability to match individuals or dogs across databases. This is perhaps reflected in Fig. 5, where there is no visual relationship between the number of dog licences and microchip registrations per year. Further, it is not possible to directly compare individual dog licences and microchipping, noting that individual dog licences are issued annually, whereas the latter are only assigned once. In addition, compliance with national legislation (on licencing and on microchipping) are uncertain, and may be relatively low. Similar challenges were seen in earlier work by Downes et al. [30, 31], leading to a focus on demographic change rather than national estimates.

The role of dog control centres in Ireland has changed substantially in recent years. During the period 2004–2020, but particularly since about 2015, the number of dogs managed through these centres has substantially decreased (Fig. 6). Further, over the last 10 years, there has been a dramatic drop in the number of dogs seized and the number of dogs euthanised or which have died of natural causes. Concurrently, there has been a more gradual decrease in the number of dogs surrendered or collected, and, since 2012, an increase in the number of dogs either sent to dog welfare organisations or reclaimed/rehomed. We speculate, but cannot confirm based on these national data, that the decreasing role of dog control centres is linked with an increasing role for dog welfare organisations. This is an area of separate study. As highlighted elsewhere, the DAFM Animal Welfare Grants have provided substantial support over some years to registered animal welfare organisations to assist in delivery of animal care and animal welfare services (Table 1) [42].

The 6-month pilot study on online dog sales was undertaken to assess the utility of these methods in contributing to an understanding of aspects of the dog population in Ireland. Our results support its usefulness. Extrapolating from the monthly mean, it is plausible that approximately 30,000 dogs were listed on these two websites over the previous 12 months. Of those dogs listed on these sites, information are now available on breed, age, location, price and microchip numbers. As expected, the majority of these dogs are puppies. With longer-term monitoring of these sites, it could be possible to assess trends in supply and demand based on price, to identify (potential changes in) favoured breeds, as well as identifying high-volume sellers. This methodology could also be considered, pending legal and ethical considerations, to support national regulatory action, including an assessment of legislative compliance.

Irish pet insurance data are currently not available for analysis. In 2017, it was suggested that ‘*the pet insurance market in Ireland is in its relative infancy* (and) *according to figures from Insurance Ireland, fewer than 10% of pets are insured here. In the UK, the equivalent figure is around 25%*’ [67]. In 2018, research conducted by Allianz Nationwide revealed that “*70% of dog owners and nearly 90% of cat owners are without pet health cover*” [68]. In 2021, a survey conducted by Pet Sitters Ireland found that “*75% of people didn’t have pet insurance*” citing cost as the main reason for not taking out cover [69]. According to one insurance provider, there was a 97% increase in the number of insurance policies taken out during the first quarter of 2021 as compared to the same period in 2020 [70]. Based on lessons from Sweden, the pet insurance database has proved particularly useful in describing aspects of national dog populations, particularly with respect to mortality and morbidity, in general and with respect to defined diseases. Egenvall et al. [13] have outlined limitations with insurance data, particularly in terms of validity and representativeness. In time, analysis of similar Irish data, if available, will prove useful. 

Based on available data, the number of outward movements of dogs from Ireland has been substantially greater than the number of inward movements of dogs into Ireland. According to the TRACES database, there were 2127 inward movement of dogs from other EU Member States and the UK in 2018-July 2021 (Table 4) compared to 53,195 (Table 3) and 2045 (Table S4 in the Supplementary material) outward movements during 2016–2020 to EEA and third countries, respectively. We acknowledge that the TRACES data only provides a partial picture of all dog movements (Table 5) and relates solely to those dog movements where certification is required. Such movements would be linked to private operators or organisations that sell or supply dogs for rehoming. However, it is these movements that are of particular interest in the context of dog welfare organisations. Considering outgoing movements from Ireland in greater detail, substantial numbers of dogs were moved during 2016–20 to the UK (41,167 dogs), Sweden (6457), Italy (1874), Germany (1583) (Table 3) and Singapore (1290) (Table S4 in the Supplementary material). Data on pet passports provide some additional information about outward movements (Table 2), however, this is limited. These data reflect the issuing of passports rather than use. In contrast to the TRACES data, pet passports are required both for commercial and non-commercial movement, and do not distinguish between those dogs leaving Ireland temporarily (for example, owners going on holidays) or permanently (dog breeding establishments selling dogs abroad). Based on the data in Table 2, the number of passports issued annually during 2014–20 has been remarkably stable, particularly in later years. With respect to inward movements, there was a marked increase, albeit from a low base, in imports from Hungary, Poland and Romania in the first 7 months of 2021 compared to each of the full calendar years of 2018, 2019 and 2020 (Table 4). We also have some access to data from commercial operators, however, this has proved difficult to assemble and interpret given that data were available for differing time periods and in different formats.

Each of the existing databases relating to dogs in Ireland needs to be interpreted with care. As highlighted in Table 5, the assessed quality of these existing databases is very variable, and often poor. None of the available data sources are of a quality that would allow a valid estimation either of the Irish pet dog population or the movement of dogs to and from Ireland (see Table 5), as to varying degrees they suffer from missing information, inconsistent data gathering mechanisms and most importantly a lack of linkage to each other. This was one of the key findings of this study. Consequently, we have refrained from presenting analytic statistics (estimates of trend, *p* values, confidence intervals etc.) throughout the manuscript as we do not believe they would be valid. Based on our qualitative assessment of these databases, confidence in the accuracy of information was only possible with the dog movement data from the European Commission (which was assessed as very high). Further, the representativeness of these databases was assessed as either unknown (the dog microchipping and identification data) or low. Relevant to this and in the context of data from dog control centres in Ireland, O’Sullivan and Hanlon [2] suggested that methods for data capture and utilisation varied considerably among Local Authorities. These authors suggest that standardisation of data capture and utilisation of dog control services would provide an opportunity to develop cohesive national policy and an improved approach to responsible dog ownership in Ireland.

The data from commercial organisations were particularly difficult to use, as these data are collected differently by different companies. It is likely that they are a conservative estimate of numbers of dogs travelling, particularly for ferry companies, given the potential for owner underreporting. We also note that no record is available of the movement of dogs across the border between Ireland and Northern Ireland. Some data from Northern Ireland is available with the council dog summary statistics [71]. In Northern Ireland, ferry companies previously provided the Department of Agriculture, Environment and Rural Affairs (DAERA) with a (conservative) estimate of 20,000 dog movements moving annually between GB and NI.

In Table 5, we present a range of suggestions to address the aforementioned data quality concerns. In particular, the linking of existing national databases (individual dog identification, dog licensing, dog control statistics) has the potential to improve both the representativeness and accuracy of information about the Irish pet dog population. We understand that this could be achieved within the existing legislative framework (that is, the legislative framework for reliable and accurate data collection already exists), as previously suggested by others, including Wedderburn [36] and Alston [72], and illustrate this proposal in Fig. 7. To illustrate, although the application form for a licence includes a place to insert the microchip number, it is very unfortunate that a microchip number is not a requirement of licensing [73]. We anticipate multiple potential beneficiaries from such a centralised database. It would contribute to the compliance and enforcement work undertaken by relevant authorities (dog wardens, port authorities, Gardaí [the Irish police]), and at relevant points of entry and exit (ports, airports) or control (rehoming centres). If these data could be accessed in real-time, this would enable authorities to identify stolen dogs, and prospective owners to cross-check the validity of information in relation to animals presented for sale. A centralised database would also facilitate communications across relevant policy areas, noting that DAFM currently has responsibility for legislation in relation to microchipping and the sale or supply of pets, whereas DRCD is responsible for the Control of Dogs Act, including licensing and strays. These challenges are not unique to Ireland. In the UK for example, concerns have been raised in relation to the recording of microchip data, where there are currently at least 16 different databases, without agreed common standards [74].

## Conclusions

This study highlights the challenges faced when using existing national data to gain insights into the dog population of Ireland. Although it was not possible to estimate the dog population of Ireland, some temporal changes are apparent. Based on national data on dog licensing and microchipping registration, pet dog numbers have remained relatively stable in recent years (ie prior to the COVID-19 pandemic). Since 2015, there has been a substantially decrease in the number of dogs managed through dog control centres, concurrent – we speculate – with an increasing role for dog welfare organisations. We note the potential utility of online private dog sales, as an additional data source to consider. Although the data are incomplete, there appear to be substantial, and increasing, number of dogs moving from Ireland to UK, Sweden, Italy, Germany and Singapore. We also note an increase (albeit much smaller) in the number of dogs being moved into Ireland. The linking of existing national databases (individual dog identification, dog licensing, dog control statistics) has the potential to improve both the representativeness and accuracy of information about the Irish pet dog population. In the next phases of our work, we will focus on the work of dog welfare organisations, given both the increased role played by these organisations and the substantial public funding that has been committed in this sector.

## Supplementary Information

**Additional file 1: Table S1**. The number of dog licences issued in Ireland during 2000-2020, by type of licence. These data were collated by the Department of Rural and Community Development and are available at https://www.gov.ie/en/collection/879d4c-dog-control-statistics/. **Table S2**. The number of dog microchips issued in Ireland by Animark, Fido and Microdog ID from 2015 to 2020 and by the Irish Kennel Club from 2015 to 2020. **Table S3**. Annual statistics relevant to dog control centres in Ireland during 2004-2020, including the number of dogs on hand at the start and end of each year, the number of incoming dogs (either surrendered/collected or seized), the number of dogs, and the number of outgoing dogs (euthanised or died from natural causes, reclaimed/rehomed or transferred to a dog welfare organisation). These data were collated by the Department of Rural and Community Development and are available at https://www.gov.ie/en/collection/879d4c-dog-control-statistics/. **Table S4**. The number of dog movements from Ireland to third countries during 2016-20, as recorded in TRACES, which is the online platform of the European Commission to facilitate sanitary and phytosanitary certification of animals, animal products, food and feed and plants, into the EU, for intra-EU trade and EU exports (https://ec.europa.eu/food/animals/traces_en). **Table S5**. The number of dogs recorded on commercial flights into Dublin airport during 2015 to June 2021. **Table S6**. The number of dogs recorded on commercial flights into Shannon airport during 2015 to June 2021. **Table S7**. The number of dogs recorded on commercial ferries into Cork Roscoff from July to October 2020. **Table S8**. The number of dogs recorded on commercial ferries into Cork Ringaskiddy from January to February 2020. **Table S9**. The number of dogs recorded on commercial ferries into Rosslare, Co. Wexford from 2018 to May 2021.

## Data Availability

Some of the datasets used in the current study are publicly available.
