# Supplementary material for: Understanding the dog population in the Republic of Ireland: insight from existing data sources?
Source: Ir Vet J. 2022 Jul 14;75:16. doi: 10.1186/s13620-022-00223-8 (PMC9281166; doi:10.1186/s13620-022-00223-8)
Supplement: Supplementary file 1 — Additional file 1: Table S1. The number of dog licences issued in Ireland during 2000-2020, by type of licence. These data were collated by the Department of Rural and Community Development and are available at https://www.gov.ie/en/collection/879d4c-dog-control-statistics/. Table S2. The number of dog microchips issued in Ireland by Animark, Fido and Microdog ID from 2015 to 2020 and by the Irish Kennel Club from 2015 to 2020. Table S3. Annual statistics relevant to dog control centres in Ireland during 2004-2020, including the number of dogs on hand at the start and end of each year, the number of incoming dogs (either surrendered/collected or seized), the number of dogs, and the number of outgoing dogs (euthanised or died from natural causes, reclaimed/rehomed or transferred to a dog welfare organisation). These data were collated by the Department of Rural and Community Development and are available at https://www.gov.ie/en/collection/879d4c-dog-control-statistics/. Table S4. The number of dog movements from Ireland to third countries during 2016-20, as recorded in TRACES, which is the online platform of the European Commission to facilitate sanitary and phytosanitary certification of animals, animal products, food and feed and plants, into the EU, for intra-EU trade and EU exports (https://ec.europa.eu/food/animals/traces_en). Table S5. The number of dogs recorded on commercial flights into Dublin airport during 2015 to June 2021. Table S6. The number of dogs recorded on commercial flights into Shannon airport during 2015 to June 2021. Table S7. The number of dogs recorded on commercial ferries into Cork Roscoff from July to October 2020. Table S8. The number of dogs recorded on commercial ferries into Cork Ringaskiddy from January to February 2020. Table S9. The number of dogs recorded on commercial ferries into Rosslare, Co. Wexford from 2018 to May 2021. [file 13620_2022_223_MOESM1_ESM.docx]

**Supplementary material**

| Table S1. The number of dog licenses issued in Ireland during 2000-2020, by type of licence. These data were collated by the Department of Rural and Community Development and are available at <https://www.gov.ie/en/collection/879d4c-dog-control-statistics/> | | | |
| --- | --- | --- | --- |
| Year | Individual dog licence  *(for one dog for a period of 12 months)*  *[€20 in 2020]* | Lifetime dog licence  *(for the lifetime of one dog)*  *[€140 in 2020]* | General dog licence  *(for owners of kennels for a period of 12 months)*  *[€400 in 2020]* |
| 2000 | 158,200 | - | 185 |
| 2001 | 155,761 | - | 203 |
| 2002 | 172,437 | - | 243 |
| 2003 | 189,356 | - | 292 |
| 2004 | 196,696 | - | 321 |
| 2005 | 195,769 | - | 391 |
| 2006^a^ | 197,779 | - | 395 |
| 2007 | 216,980 | - | 440 |
| 2008 | 214,724 | - | 466 |
| 2009 | 212,867 | - | 426 |
| 2010 | 210,049 | - | 407 |
| 2011 | 215,241 | - | 385 |
| 2012 | 188,910 | 282 | 229 |
| 2013 | 188,702 | 316 | 256 |
| 2014 | 190,833 | 313 | 167 |
| 2015 | 196,956 | 317 | 168 |
| 2016 | 197,920 | 548 | 169 |
| 2017 | 199,710 | 814 | 234 |
| 2018 | 204,924 | 2,857 | 243 |
| 2019 | 212,559 | 4,378 | 255 |
| 2020 | 200,857 | 6,713 | 239 |
| Total | 4,117,230 | 16,538 | 6,114 |

| Table S2. The number of dog microchips issued in Ireland by Animark, Fido and Microdog ID from 2015 to 2020 and by the Irish Kennel Club from 2015 to 2020 | | | | | | | |
| --- | --- | --- | --- | --- | --- | --- | --- |
| Company | 2015 | 2016 | 2017 | 2018 | 2019 | 2020 | Total |
| Animark | 7,297 | 37,906 | 21,445 | 18,389 | 17,619 | 27,588 | 147,407 |
| Fido | 22,934 | 105,329 | 43,381 | 37,577 | 35,255 | 41,547 | 295,627 |
| Irish Kennel Club | - | - | 14,334 | 14,954 | 14,899 | 16,014 | 70,533 |
| Microdog ID | 5,624 | 23,443 | 13,030 | 12,999 | 12,179 | 9939 | 81004 |
| Total | 35,855 | 166,678 | 92,190 | 83,919 | 79,952 | 95,088 | 594,571 |

| Table S3. Annual statistics relevant to dog control centres in Ireland during 2004-2020, including the number of dogs on hand at the start and end of each year, the number of incoming dogs (either surrendered/collected or seized), the number of dogs, and the number of outgoing dogs (euthanised or died from natural causes, reclaimed/rehomed or transferred to a dog welfare organisation). These data were collated by the Department of Rural and Community Development and are available at <https://www.gov.ie/en/collection/879d4c-dog-control-statistics/> | | | | | | | |
| --- | --- | --- | --- | --- | --- | --- | --- |
| Year | On-hand (end of previous year) | Incoming dogs | | Outgoing dogs | | | On-hand (end of year) |
|  |  | Surrendered / Collected | Seized | Euthanised / Death by natural causes | Reclaimed / Rehomed | Transferred to dog welfare organisations |  |
| 2004 | 144 | 14,823 | 9,749 | 16,598 | 7,939 | - | 178 |
| 2005 | 178 | 15,078 | 10,254 | 16,546 | 8,818 | - | 146 |
| 2006 | 148 | 16,338 | 7,825 | 14,598 | 9,539 | - | 203 |
| 2007 | 210 | 15,587 | 7,723 | 12,649 | 10,666 | - | 205 |
| 2008 | 202 | 12,707 | 7,942 | 10,094 | 10,561 | - | 196 |
| 2009 | 185 | 9,568 | 6,845 | 6,536 | 9,921 | - | 141 |
| 2010 | 145 | 11,638 | 5,257 | 5,987 | 10,889 | - | 174 |
| 2011 | 174 | 11,904 | 6,182 | 6,229 | 11,772 | - | 259 |
| 2012 | 259 | 11,084 | 6,921 | 5,011 | 6,841 | 6,150 | 262 |
| 2013 | 262 | 9,309 | 6,734 | 4,048 | 5,969 | 6,124 | 242 |
| 2014 | 242 | 8,941 | 6,050 | 3,203 | 5,402 | 6,403 | 225 |
| 2015 | 225 | 11,757 | 1,660 | 2,095 | 5,230 | 6,068 | 253 |
| 2016 | 252 | 12,661 | 172 | 1,738 | 4,949 | 6,180 | 219 |
| 2017 | 218 | 11,577 | 197 | 1,052 | 4,807 | 5,938 | 195 |
| 2018 | 199 | 9,737 | 224 | 824 | 4,343 | 4,749 | 244 |
| 2019 | 244 | 8,888 | 274 | 440 | 4,140 | 4,649 | 177 |
| 2020 | 175 | 5,112 | 198 | 199 | 2,627 | 2,545 | 114 |
|  | | | | | | | |

| Table S4. The number of dog movements from Ireland to third countries during 2016-20, as recorded in TRACES, which is the online platform of the European Commission to facilitate sanitary and phytosanitary certification of animals, animal products, food and feed and plants, into the EU, for intra-EU trade and EU exports (<https://ec.europa.eu/food/animals/traces_en>) | | | | | | |
| --- | --- | --- | --- | --- | --- | --- |
| Country | 2016 | 2017 | 2018 | 2019 | 2020 | Total |
| Argentina | - | - | 1 | 2 | 1 | 4 |
| Australia | 22 | 3 | 46 | 84 | 36 | 191 |
| Bahrain | - | - | 6 | 1 | - | 7 |
| Barbados | - | - | - | 1 | - | 1 |
| Bermuda | - | - | 6 | 1 | - | 7 |
| Brazil | - | - | 14 | 49 | 26 | 89 |
| Canada | - | 4 | 17 | 4 | 2 | 27 |
| Cayman Islands | - | - | 3 | 1 | - | 4 |
| Chile | - | 1 | - | - | - | 1 |
| China | 9 | - | 6 | 1 | 1 | 17 |
| Dominican Rep | - | - | 1 | - | - | 1 |
| Dubai | - | - | - | - | 4 | 4 |
| Egypt | - | - | - | 1 | - | 1 |
| Hawaii | - | - | - | 2 | - | 2 |
| Hong Kong | - | 1 | 9 | 3 | 9 | 22 |
| India | - | - | 1 | 2 | - | 3 |
| Israel | - | - | 3 | 2 | 3 | 8 |
| Jamaica | - | - | - | 1 | - | 1 |
| Japan | - | 1 | 1 | 4 | 1 | 7 |
| Jordan | - | - | - | 1 | - | 1 |
| Kenya | - | - | 2 | - | - | 2 |
| Kuwait | - | - | - | 1 | - | 1 |
| Laos | - | - | 1 | - | - | 1 |
| Mauritius | - | - | 2 | 3 | - | 5 |
| Mexico | 1 | - | - | - | 2 | 3 |
| New Zealand | - | - | 11 | 15 | 4 | 30 |
| Oman | - | - | 2 | - | - | 2 |
| Panama | - | - | 1 | 1 | - | 2 |
| Philippines | - | - | - | 1 | - | 1 |
| Puerto Rico | 1 | - | - | - | - | 1 |
| Qatar | - | - | - | 2 | - | 2 |
| Russia | - | - | 2 | 1 | 1 | 4 |
| Saudi Arabia | - | 1 | - | 3 | - | 4 |
| Sierra Leon | - | - | 1 | - | - | 1 |
| Singapore | 1 | 1 | 72 | 271 | 945 | 1,290 |
| South Africa | - | 3 | 11 | 6 | 12 | 32 |
| South Korea | - | 1 | - | 1 | 2 | 4 |
| St Lucia | - | - | 1 | - | - | 1 |
| Thailand | - | - | 1 | 2 | 1 | 4 |
| Turkey | - | - | - | - | 1 | 1 |
| Ukraine | - | - | 2 | - | - | 2 |
| United Arab Emirates | 2 | 3 | 23 | 13 | 8 | 49 |
| USA | 2 | - | 6 | 10 | 12 | 30 |
| Zambia | - | 1 | - | - | - | 1 |
| Unclear | 39 | 128 | - | - | 7 | 174 |
| Total | 77 | 148 | 252 | 490 | 1,078 | 2,045 |

A summary of incoming dog movements based on data from commercial companies

a. Airlines

| Figure S5. The number of dogs recorded on commercial flights into Dublin airport during 2015 to June 2021 | | | | | | | | |
| --- | --- | --- | --- | --- | --- | --- | --- | --- |
| Country | 2015 | 2016 | 2017 | 2018 | 2019 | 2020 | 2021  to June | Total |
| European Union Countries |  |  |  |  |  |  |  |  |
| Austria | 2 | - | - | - | 1 | - | - | 3 |
| Belgium | - | - | - | 1 | - | - | 2 | 3 |
| Bulgaria | 2 | - | - | - | - | - | - | 2 |
| Croatia | 1 | - | - | - | - | - | - | 1 |
| Cyprus | 1 | - | - | - | - | 2 | - | 3 |
| Czech Republic | 1 | - | - | - | - | - | - | 1 |
| Finland | - | 1 | - | - | 1 | - | - | 2 |
| France | 1 | 4 | - | - | - | 1 | - | 6 |
| Germany | 1 | - | - | 1 | - | - | - | 2 |
| Italy | 1 | 1 | - | 1 | - | - | - | 3 |
| Malta | 2 | 1 | - | - | - | - | - | 3 |
| Netherlands | 3 | 3 | - | 1 | - | 1 | 2 | 10 |
| Poland | - | - | - | - | 1 | - | - | 1 |
| Portugal | - | 1 | 1 | - | 3 | 3 | 2 | 10 |
| Romania | 16 | 14 | 1 | 1 | 14 | 6 | 2 | 54 |
| Spain | 3 | - | - | 1 | 1 | 4 | 7 | 16 |
| Non-European Union Countries |  |  |  |  |  |  |  |  |
| Afghanistan | - | - | - | - | - | 1 | - | 1 |
| Angola | - | - | 1 | - | - | - | - | 1 |
| Argentina | 2 | 2 | 2 | - | 9 | 2 | 6 | 23 |
| Ascension Island | - | - | - | - | - | 1 | - | 1 |
| Australia | 135 | 177 | 182 | 94 | 137 | 125 | 53 | 903 |
| Bahrain | - | - | 1 | - | 2 | 4 | - | 7 |
| Belarus | - | - | - | - | - | 1 | - | 1 |
| Bermuda | - | - | - | - | 2 | 1 | - | 3 |
| Bosnia & Herzegovina | - | - | - | - | - | - | 1 | 1 |
| Brazil | 2 | 1 | 2 | 17 | 72 | 49 | 30 | 173 |
| Cameroon | - | - | - | - | - | 1 | - | 1 |
| Canada | 70 | 64 | 79 | 82 | 157 | 113 | 35 | 600 |
| Cayman Islands | - | - | - | 1 | - | - | - | 1 |
| Chile | 1 | - | - | 1 | 2 | 4 | - | 8 |
| China | - | 2 | 3 | 2 | 10 | 3 | - | 20 |
| Colombia | - | - | - | 1 | 2 | - | 3 | 6 |
| Costa Rica | - | - | 2 | - | 1 | - | - | 3 |
| Curacao | - | - | - | 1 | - | - | - | 1 |
| Dominican Republic | - | - | 4 | - | - | - | - | 4 |
| Ecuador | 1 | - | - | - | - | - | - | 1 |
| Egypt | 1 | 1 | - | - | - | 1 | - | 3 |
| Georgia | - | - | - | - | 2 | - | - | 2 |
| Guatemala | - | - | - | 1 | - | 1 | 2 | 4 |
| Hong Kong | 2 | - | - | 1 | 11 | 6 | 34 | 54 |
| Iceland | - | - | - | - | 2 | - | - | 2 |
| India | 1 | 1 | - | - | 5 | 2 | 2 | 11 |
| Indonesia | - | - | 3 | 3 | - | 2 | - | 8 |
| Israel | 3 | 1 | 2 | 1 | 9 | 4 | 3 | 23 |
| Japan | - | - | - | - | 2 | - | - | 2 |
| Jordan | 2 | - | 2 | - | 4 | 1 | 2 | 11 |
| Kenya | 1 | - | - | - | - | 2 | 1 | 4 |
| Kosovo | - | 1 | - | - | - | - | - | 1 |
| Kuwait | - | 3 | - | 2 | 1 | 1 | - | 7 |
| Kyrgyzstan | - | - | - | - | 1 | - | - | 1 |
| Lebanon | - | - | 1 | 1 | 2 | 5 | 2 | 11 |
| Macedonia | - | 1 | - | - | - | - | 3 | 4 |
| Malawi | 1 | - | - | 1 | - | 1 | 1 | 4 |
| Malaysia | - | 4 | - | - | 3 | 4 | 1 | 12 |
| Mauritius | 1 | - | 2 | - | 1 | - | - | 4 |
| Mexico | 2 | 5 | - | 2 | 7 | 4 | 4 | 24 |
| Moldova | - | - | - | - | 4 | 1 | - | 5 |
| Morocco | - | - | - | - | 2 | - | - | 2 |
| Namibia | - | - | - | - | 5 | - | - | 5 |
| Nepal | - | - | - | - | - | 1 | - | 1 |
| New Zealand | 12 | 17 | 19 | 19 | 22 | 10 | 5 | 104 |
| Nigeria | - | 2 | - | - | - | 1 | - | 3 |
| Oman | - | - | 1 | 2 | 6 | 4 | - | 13 |
| Panama | - | - | 1 | 1 | 1 | - | - | 3 |
| Papua New Guinea | - | - | - | - | 1 | - | - | 1 |
| Peru | - | - | - | 2 | - | - | - | 2 |
| Philippines | 1 | 1 | 3 | - | 3 | 3 | 2 | 13 |
| Puerto Rico | - | - | 1 | - | - | - | - | 1 |
| Qatar | 3 | 3 | 4 | 6 | 20 | 15 | 5 | 56 |
| Russia | 5 | - | 1 | 1 | 21 | 6 | 8 | 42 |
| Saudi Arabia | 1 | - | - | 1 | 2 | 2 | - | 6 |
| Serbia | - | 1 | 1 | - | 1 | - | 1 | 4 |
| Sierra Leone | - | - | - | - | - | 1 | - | 1 |
| Singapore | 1 | - | - | 1 | - | - | 3 | 5 |
| Sint Maarten | - | - | - | - | - | - | 2 | 2 |
| South Africa | 11 | 5 | 5 | 4 | 56 | 26 | 20 | 127 |
| South Korea | 1 | - | 2 | - | 5 | - | 2 | 10 |
| Taiwan | - | - | - | 2 | 1 | 1 | - | 4 |
| Tanzania | - | - | - | - | 3 | 2 | - | 5 |
| Thailand | 4 | 1 | 2 | 2 | 1 | 7 | 1 | 18 |
| Turkey | 5 | 4 | 5 | 5 | 11 | 8 | 7 | 45 |
| Uganda | - | - | - | - | 1 | - | 1 | 2 |
| Ukraine | 1 | 1 | - | 2 | 3 | 1 | 1 | 9 |
| United Arab Emirates | 26 | 40 | 37 | 43 | 47 | 84 | 13 | 290 |
| United Kingdom^a^ | - | - | - | - | 1 | - | 9 | 10 |
| USA | 309 | 369 | 462 | 520 | 726 | 581 | 178 | 3,145 |
| Uzbekistan | - | - | - | - | 1 | - | - | 1 |
| Venezuela | - | - | - | 1 | 1 | - | - | 2 |
| Vietnam | - | - | 1 | 1 | 2 | 1 | - | 5 |
| Zambia | - | - | 2 | 1 | 4 | 1 | - | 8 |
| Zimbabwe | 2 | 4 | - | 1 | - | 2 | - | 9 |
| Total | 641 | 736 | 835 | 832 | 1,416 | 1,114 | 456 | 6,030 |
| a. The United Kingdom left the European Union on 31 January 2020 | | | | | | | | |

| Figure S6. The number of dogs recorded on commercial flights into Shannon airport during 2015 to June 2021 | | | | | | | | |
| --- | --- | --- | --- | --- | --- | --- | --- | --- |
| Country | 2015 | 2016 | 2017 | 2018 | 2019 | 2020 | 2021 to June | Total |
| European Union Countries |  |  |  |  |  |  |  |  |
| Belgium | - | - | - | 1 | - | - | - | 1 |
| France | - | - | 2 | 2 | - | - | - | 4 |
| Germany | - | - | - | - | 1 | - | 1 | 2 |
| Spain | - | - | - | - | 1 | 1 | - | 2 |
| Non-European Union Countries |  |  |  |  |  |  |  |  |
| Canada | - | - | - | 1 | - | - | - | 1 |
| Mexico | 1 | 1 | - | - | - | - | - | 2 |
| Switzerland | - | - | - | - | 1 | 1 | - | 2 |
| United Kingdom^a^ | - | - | - | - | - | - | 1 | 1 |
| USA | 10 | 11 | 25 | 26 | 46 | 8 | - | 126 |
| Total | 11 | 12 | 27 | 30 | 49 | 10 | 2 | 141 |
| a. The United Kingdom left the European Union on 31 January 2020 | | | | | | | | |

b. Ferry companies

| Figure S7. The number of dogs recorded on commercial ferries into Cork Roscoff from July to October 2020 | | |
| --- | --- | --- |
| Country | July to October 2020 | Total |
| European Union countries |  |  |
| Belgium | 3 | 3 |
| Czech Republic | 1 | 1 |
| France | 19 | 19 |
| Germany | 8 | 8 |
| Hungary | 1 | 1 |
| Ireland | 105 | 105 |
| Malta | 1 | 1 |
| Netherlands | 4 | 4 |
| Portugal | 1 | 1 |
| Romania | 2 | 2 |
| Slovakia | 1 | 1 |
| Spain | 5 | 5 |
| Switzerland | 4 | 4 |
| Non-European Union countries |  |  |
| United Kingdom* | 18 | 18 |
| Total | 173 | 173 |
| a. The United Kingdom left the European Union on 31 January 2020 | | |

| Figure S8. The number of dogs recorded on commercial ferries into Cork Ringaskiddy from January to February 2020 | | |
| --- | --- | --- |
| Country | January to February 2020 | Total |
| European Union countries |  |  |
| Ireland | 35 | 35 |
| Portugal | 5 | 5 |
| Spain | 18 | 18 |
| Non-European Union countries |  |  |
| United Kingdom^a^ | 5 | 5 |
| Total | 63 | 63 |
| a. The United Kingdom left the European Union on 31 January 2020 | | |

| Figure S9. The number of dogs recorded on commercial ferries into Rosslare, Co. Wexford from 2018 to May 2021 | | | | | |
| --- | --- | --- | --- | --- | --- |
| Ferry company | 2018 | 2019 | 2020 | 2021 to May | Total |
| Brittany Ferries | - | - | 344 | 213 | 557 |
| Irish Ferries | 904 | - | - | - | 904 |
| Stena Line | 2,361 | 2,141 | 1,603 | 1,235 | 7,340 |
| Total | 3,265 | 2,141 | 1,947 | 1,448 | 8,801 |
